# Exploring MAR perceptions in Kosovo – disparities in knowledge about MAR among young people

**DOI:** 10.12688/openreseurope.18974.2

**Published:** 2025-05-28

**Authors:** Vera Dimitrievska, Jana Meloska, Virginie Rozée, Manon Vialle, Kristien Hens, Joke Struyf, Anna De Bayas Sanchez, Michaela Fuller Fuller, Francisco Güell, María López-Toribio, José Miguel Carrasco

**Affiliations:** 1 Research Department, HeatlhGrouper, Skopje, North Macedonia, Skopje, 1000, North Macedonia; 2 University of American College, Skopje, North Macedonia; 3 INED, Research Unit “Sexual and reproductive Health and Rights”, Aubervilliers, France; 4 University of Antwerpen, Department of Philosophy, Antwerpen, Belgium; 5 Medistella Mediversvm s.r.o., Management, Prague, Czech Republic; 6 Institute for Medical Humanities 1st Faculty of Medicine of Charles University, Prague, Czech Republic; 7 University of Navarra, ICS, Pamplona, Spain; 8 Universidad Anáhuac México, Facultad de Bioética, Ciudad de México, Mexico; 9 APLICA Investigación y Traslación Cooperative, Madrid, Spain

**Keywords:** Parenthood, (in)fertility, MAR techniques, perceptions, young people, Kosovo

## Abstract

Research on knowledge and perception about (in)fertility and Medically Assisted Reproduction (MAR) techniques among young people in Kosovo is lacking. Part of the European Project’s B²-InF’s team conducted a qualitative study in Kosovo with young adults (18–30) in 2021 assessing perceptions and views related to MAR and information provided by the MAR clinics in Kosovo. Main objective of this study is to explore perceptions about motherhood/fatherhood and to understand (in)fertility issues among young people in Kosovo. It is focused to delve more into MAR knowledge and techniques of young people in Kosovo and in general. Fifteen interviews with young people living in Kosovo between June and July 2021 were conducted. A total of 3 themes emerged during the qualitative analysis and were grouped in three main theme levels. The first level, perceptions about motherhood and fatherhood among young people in Kosovo is about different views of young people in Kosovo, desires about becoming parents, what is their personal attitude towards parenting. The second, relevance/importance of fertility in Kosovo context is looking at importance of (in)fertility within the young people from gender perspective. The third, MAR perceptions and new techniques that are prevailing in the global sense as different access opportunities are present. The findings from our interviews demonstrated that parenthood is still gender related. Male (in)fertility is often considered a taboo among the young people in Kosovo. A substantial amount of lack of knowledge was found in relation to MAR techniques, highlighting the importance of detailed information about the whole process of MAR treatments.

## Background

Over the past decades the research about parenthood/motherhood, fertility and treatments about infertility gained an immense attention in research and online forums. The research studies show that people view being a parent as an essential aspect of their life cycle ( Daniluk, 2001). After seeing a substantial decline of fertility rate levels in entire Europe in the last decades also the parental age started to increase ( Regushevskaya *et al.*, 2013; Schmidt *et al.*, 2012). This trend affected also the countries from Western Balkan during the 1990s in a way of fewer children born per couple in different age from different reasons. The main reasons are cultural and economic factors. From one point of view, there have deep-rooted traditions, the predominance of conservative family values within a predominant Muslim population, a low acceptance of birth control and modern contraception, high marriage rates, and low economic development. On another point of view, there is an unclear and generally constant pessimistic political situation, persistent agrarian settlement, poor female education, and low female economic activity ( Čipin *et al.*, 2020; Frejka & Gietel-Basten, 2016)

Number of studies have demonstrated that increasing of parental and maternal age is associated with extended gestation period, and with consequences of pregnancy, like miscarriage ( Archer *et al.*, 2007; Cleary-Goldman *et al.*, 2005; Sartorius & Nieschlag, 2010).

The (in)fertility patterns affected the Western Balkan countries in the late 90s. Demographic structure of fertile rates levels among women in this region has changed drastically. While in 1991 the children on average per woman was 3.25 ( Brunborg, 2002), in 2019, Kosovo had 1.97 children on average per woman, which was higher than the average fertility rate in Europe. The average mother's age for her first childbirth this year was 29 ( World Bank, 2023). Postponing family formation compared to Western Europe countries is slowly declining especially in Kosovo due to influence of various factors such as cultural, traditional, political and ideational aspects ( Lerch, 2018). This fertility behaviour is argued in literature that societal context played major role. The influence of religious discourses and government measures of pro-natal policies where women’s role and traditional values in upbringing the child are extensively emphasized ( Shiffman *et al.*, 2002). Moreover, Kosovo is well-known for its secular past of customary laws and mainstreamed social systems, which have helped traditional relationship structures to developed ( Kaser, 2008). Tradition and religion are still intertwined together to become a single entity in some parts. Although their objectives are slightly different, tradition promotes as many families as possible since it is a symbol of its power, while the religious values having as many members as possible ( Krasniqi, 1990).

A few studies looked at the gender aspects and fertility issue in Kosovo ( Predojević, 2001; Pushka, 1989). Women's inferior status within the family and society was largely determined by their lack of agency in decision-making and their acceptance of the necessity of their current relationships. According to Predojević (2001) a woman in rural areas still obeys to her husband's will and has little options to further her lower-level education. She was forced to choose between total subjugation and the inability to alter his standing in the community as a result, trapping herself in an endless cycle. She thereby assumes the role of "guardian of tradition," ensuring the continuation of the patriarchal lifestyle despite the fact that it is based on principles that are turned against women ( Latifi, 1999; Pushka,1989).

Despite the steady fertility rates in Kosovo and all reforms, changes have embarked in the recent years. MAR industry is also present in Kosovo, it counts 5 private MAR clinics. These clinics are highly focused on describing step-by-step procedures and medical indications of treatments, Medical Assisted Reproduction (MAR) techniques have been legally regulated in 2006 based on the societal context, including the clinical and biological procedures.

Most of the information referred to in the legal framework are aimed for heterosexual couples. Surrogacy is prohibited in Kosovo as an alternative way of establishing a family. People in same-sex relationships are still legally barred from marrying and starting a family.

## Methods

Part of the European Project B²-InF team conducted a qualitative study in Kosovo with young adults (18–30) in 2021 assessing sociocultural, gender and legal perspectives related to MAR and information provided by the MAR clinics. The study employed the semi-structured interview technique for the qualitative data collection. The B²-InF team carried out and analyzed a total of 15 interviews with young people living in Kosovo. Interviews lasted between 40 and 55 min, following a script agreed by the research consortium, in order to explore perceptions among young people about parenthood, (in)fertility and MAR perceptions techniques available in Kosovo. Primary criteria to select participants were heterogeneous young people from 18–30 of age ( Table 1).

**Table 1.** Demographic characteristic of participants.

	Participants (N=15)	
	N	%
**Age**		
18–22	8	53
23–26	6	40
27–30	1	7
**Gender**		
Male	8	53
Female	6	40
Transgender male to female	1	7
**Sexual orientation**		
Heterosexual	13	86
Homosexual	1	7
Bisexual	1	7
**Occupation**		
Student	2	14
Employed	8	53
Unemployed	5	33
**Education**		
Secondary school	1	7
High school	9	60
Bachelor degree	5	33
**Residence**		
Urban area	8	53
Semi-urban area	3	20
Rural area	4	27
**Relationship**		
Yes	8	53
No	7	47
**Marital status**		
Single	10	67
Married	5	53
**Religious**		
Christian	1	7
Jewish	0	0
Muslim	13	86
Others	1	7

The study used purposive sampling technique in order to capture heterogeneity of young participants (gender, age, residence, marital status/relationship, sexual orientation, education and religion). Majority of the participants in the interviews were recruited primarily from Prishtina, the capital city of Kosovo, but some interviewees came from other areas outside the capital. A thematic analysis was performed using Atlas.ti software (version 23.2.1).

For the purpose of this study, we used an exploratory approach in order to understand better what the perceptions with young people in Kosovo are and to fill the gap in the literature about the motherhood/fatherhood trajectory itself. There remains a lack of knowledge about (in)fertility relevance in Kosovo and MAR perceptions within the young people. Therefore, this study provides an opportunity to gain insight into fertility behaviour and reproduction as they capture views among young people who are not parents about motherhood/fatherhood and in(fertility). Furthermore, it also explores the knowledge and perceptions of young people about MAR techniques in Kosovo and in general.

This project (n°2021.004) received ethical approval from the Research Ethics Committee of the University of Navarra on 29 January 2021. All interview participants provided informed consent. For in-person interviews, consent forms were signed physically, while for online interviews, the forms were sent as PDFs and returned signed by participants. The consent form indicated that transcripts would be used for preparing scientific articles and reports related to B2-InF. Additionally, participants provided audio-recorded consent at the beginning of each interview.

## Analysis of data

All interviews were conducted in the native Albanian language, recorded on audio, transcribed and translated to English, and then used pre-defined codes and validated by all consortium partners. The researcher selected a code tree containing themes and subthemes. Codes were then applied to each interview using Atlas.ti software. The local researcher independently coded the transcripts as follows. It has developed an initial code structure from transcripts of the interviews. Using this final version of the code structure, the researcher independently coded all transcripts, then discussed discrepancies, achieving consensus and assigning codes to observations by a negotiated process with the consortium members. In the next phase, we presented the themes to the young participants in order to ensure that they were, from their perspective, consistent with the viewpoint of young people. To uncover emergent themes for each participant, the next stage entailed aggregating these initial themes, which elevated the analytical process to a higher degree of abstraction. At this stage we organized the themes that had been derived inductively. A total of 3 themes emerged during coding and were grouped in three main theme levels. The first level, perceptions about motherhood and fatherhood among young people in Kosovo is about different views of young people in Kosovo, desires about becoming parents which is their personal attitude towards parenting. The second, relevance/importance of fertility in Kosovo environment context is looking at importance of (in)fertility within the young people from gender perspective. The third, MAR perceptions and new techniques that are prevailing in the global sense as different access opportunities are present. The themes are described below.

## Results

### Perceptions about motherhood and fatherhood among young people in Kosovo

The theme fatherhood/motherhood perceptions among young people in Kosovo is linked to planning, differentiation, and desire to become parentsThese issues were key across the interviews. Planning the parenthood participants is considered when they become fully grown persons, having enough financial and social stability to upbring children. With most of the participants we observed that parenthood is seen as a primary role or something that is a priority in their life. Desire to become parents were present across the interviews and strong positive attitudes towards parenthood and motherhood. Perceptions about planned parenthood are considered as a responsible feature for a future parent, which is linked to the belief that it is very important to be mature enough before becoming a parent.

*“Parenthood is a big responsibility but is a right that can’t be denied to no one. So everyone that considers themselves to be ready to offer the love, care and take the responsibility to be a parent then no one can deny the right to anyone to do that.”* (KOS 04, 30 years, transgender female to male, homosexual, Muslim)

Participants also attributed a concern to irresponsible parenting especially among parents at young age like from 17–22 years, since people in Kosovo become parents at young age. Nevertheless, they do believe that unplanned pregnancy may become a basis for certain mental health problems like depression or anxiety. On the same note, participants stated that being financially secured is an important aspect of raising children but also on the other hand it was expressed that some couples do not have access to abortion or knowledge about planning a pregnancy.

*“Because when they are not ready financially, psychologically and physically, if so I think it’s better not to have children.”* (KOS 08, 25 years, female, heterosexual, Muslim)

A highly prominent feature emphasized by the participants was differentiation among motherhood vs. fatherhood. There appeared to be a difference in perceptions about parenthood and participants believe that bigger burden falls to mothers/females, because they consider that the mothers have a stronger connection with their children as a result of the linkage with the psychological process during pregnancy, birth/delivery, breastfeeding and the hormonal changes.

*“The child's connection with the mother is stronger than with the father, not that with the father it is not a strong connection but with the mother it is much stronger in my opinion and at the same time the mother's connection with the child is stronger because she keeps it for 9 months in the womb.”* (KOS 14, 21 years, male, heterosexual, Muslim)

In relation to parenthood, participants believe that mother’s role is more demanding in the whole process of taking care of the children. Mothers carry a burden to raise the child in the intensive motherhood according to participants, which is also linked to care, education and organizing the household, because they believe that women are more capable for it than men. Participants reflected that men are expected to provide financial support or are considered as a breadwinner in general.

*“For fathers’ parenthood is seen as something that they should provide financially and for mothers it has to do a lot more with the education aspect of the children.”* (KOS 03, 20 years, male, heterosexual, None)

Social pressure to become parents plays considerable role among young people in Kosovo. Participants felt being more controlled by the society to be a parent as it is relevant for their social setting. Although many participants acknowledge that parenting is an individual choice, there is a social pressure to become parent. A few participants stated that they do not choose consciously to have children, but they are led by the expectations from their older generations. Associated with this, they reported obligation to be a parent is perceived in various ways, like to care for the child as duty, from religious view it is something that child would bring something into the life and increasing the birth rate in the country.

*“Because exactly from the social pressure they end up being a parent to continue the cycle of life and descendants. They don’t really feel the want to become a parent or are really ready psychologically or emotionally to be one.”* (KOS 03, 20 years, male, heterosexual, None)

*“For Muslim people I believe it has a lot of importance because everyone that is a Muslim want to have descendants and it is really important for our community and Muslims to have kids and continue the religion and everything.”* (KOS 05, 21 years, female, heterosexual, Muslim)

Participants have shared their opinions that society expects for everyone to have children as that is perceived as a norm, and not having children can be seen as a form of a taboo. This pressure is even more emphasised in women, as women are perceived as a means of reproduction. Majority of participants locate the pressure for having children in the religion.

*“The traditional lifestyle becomes so powerful that people don’t decide things, but just do them routinely”. (KOS 09,* 26 years, female, heterosexual, Muslim *)*

### Relevance/Importance of (in)fertility in Kosovo environment

Interviews show that young people understand the meaning of (in)fertility and perceive it as an important issue, as most of them believe they are fertile and hope to have children of their own. However, the topics like sexuality, fertility, women’s body are less discussed. While inability of being fertile among both male and female participants stated that is a curse from a God, particularly for women. Therefore, women who are childless feel more societal pressure and judged by close networks of people even further regardless if she is voluntarily or involuntarily childless. General perception from the young people in Kosovo is that women are described as ‘reproductive machines’. Participants do not believe that women after 30s will affect her ability to conceive; rather, they view fertility as a gift as long as a woman is capable of bearing children. Several participants have emphasized that women are more frequently affected with infertility compared to males.

*“Being fertile is one of the most important things, me as a woman that wants to have three or four children, depends on how will God bless me, for me it is really important to be fertile and not to have to use other alternative methods to create a family.”* (KOS 09, 26 years, female, heterosexual, Muslim)

*“I think most of the times females are the ones who have the most problems with infertility, those who have problems with the uterus as much as I have heard or read. It is the female who has the most difficulty.”* (KOS 02, 20 years, male, heterosexual, Muslim)

Male (in)fertility is often considered a taboo among the young people in Kosovo and not much discussed in public. Since this topic is hardly discussed, then understanding is lacking, particularly in rural regions, in addition to a few participants expressing extreme viewpoints and misunderstanding about male infertility. However, they are expected to procreate and carry on the family name, so men are also believed to experience infertility. Sometimes a man would prefer to undergo and seek a divorce and marry another woman than to get fertility test. Men who are infertile are perceived as being weaker and having lost their male ego.

*“Men […. ] add more people to the family, men carry this responsibility in our society”* (KOS 03, 20 years, male, heterosexual, None).

Even if they are infertile, men are expected to hide their vulnerability and sensitivity, since they are not allowed to do so. Because of the male infertility being taboo, women are consistently held accountable of their infertility.

Many participants agreed that infertility is increasing where men and women struggle equally for being infertile but in different ways. Women who are infertile, according to the participants are not able to realize their role as a mother successfully. Moreover, women are more emotional and sensitive than men with a strong desire to create a family. As for the possible causes of infertility, participants have pointed out various possible causes. Many of them agreed that age is an important factor for infertility, and various intrinsic and extrinsic factors have been mentioned by two participants, such as stress, healthy lifestyles, genetics, anatomy of female genital organs, genital infections, environmental and air pollution. Knowledge about potential reasons for (in)fertility are missing.

*“For me it is really important to have children then of course infertility would not let me accomplish my need and want to have children that I feel.”* (KOS 08, 25 years, female, heterosexual, Muslim)

*“For a person to be fertile they must first have a healthy life and not have too much stress in life, and I strongly believe that the majority of the population is fertile, but this also depends on environmental pollution, air pollution, or how people live their life, it also depends genetically.”* (KOS 13, 26 years, female, heterosexual, Muslim)

### Medical Assisted Reproduction (MAR) perceptions – what people think about it

We discussed general knowledge and personal perceptions about MAR with the participants. Overall, participants were not very familiar with the techniques available in general and those available in Kosovo, but most of them knew or at least have heard about IVF. Several participants could identify some other techniques as well, but many of them acknowledged that the procedures and the process for MAR techniques are not known, and they practically don’t know anything about it. As far as success rates were concerned, most of participants believe MAR techniques are pretty much successful. When asked whether they would undergo some sort of MAR intervention, in case it was needed, we received heterogeneous responses. One stated they would undoubtedly undergo whatever intervention is needed, while others were more reserved and replied they would prefer some more conservative treatments or an adoption as a method. Their main concerns with MAR were lack of information and anxiety about them, concerns over possible defects or syndromes or transmission of infections to the baby.

*“No, I do not have information about these services. I, as 99% of the population of Kosovo, am not informed about these things.”* (KOS 07, 21 years, male, heterosexual, Muslim)

*“… No way for artificial methods, or with sperm donation. If there is any treatment that makes me or my partner fertile, depending on who is infertile, then YES. Otherwise, adoption would be the option.”* (KOS 14, 21 years, male, heterosexual, Muslim)

*“Yes, if I would want to have a kid and I wouldn’t be able to have one then without a doubt I would try these techniques.”* (KOS 05, 21 years, female, heterosexual, Muslim)

*“If something goes wrong the child can be born with defects, this is my only concern. That the child could have defects or the child could be born with any kind of syndrome.”* (KOS 09, 26 years, female, heterosexual, Muslim).

Within the interviews we wanted to hear participants’ views about the accessibility of the MAR clinics. Some disagreeing opinions were brought out, as some believe that MAR may pose significant financial burden to potential clients, while others say that these interventions are practically free. Some participants also believe candidates for MAR should be initially assessed and fulfil certain requirements. Majority of participants have stressed that the biggest problem in accessing such services is financial incapacity of the population, as these services are not covered by the health insurance and should be paid out of pocket. Also, they have pointed out that these services are offered in limited number of clinics and that there is generally low confidence in health services provided in Kosovo. They have pointed out the low awareness in general public about the issue of MAR as an additional factor adversely influencing the accessibility of these services.

*“I think people with good financial income have the easiest access to these techniques and families that have knowledge related to these techniques.”* (KOS 08, 25 years, female, heterosexual, Muslim)

*“In Kosovo there is a lack of clinics and places that offer these opportunities.”* (KOS 11, 22 years, female, bisexual, Muslim))

*“I think it is not easy because most hesitate to ask and search for these kinds of treatments and services because it is normalized. Second the fact that there is no health insurance it makes it unaffordable for the most and third and most important as I mentioned first that for someone to look up for a service this service should be more talked about by the society.”* (KOS 04, 30 years, transgender female to male, homosexual, Muslim)

Another aspect of MAR in Kosovo during the interviews was the legal issues and what is the general knowledge. A frequent perception voiced by the young people was that they were not familiar if there is a distinct law that regulates this matter. We therefore asked about what their expectations were that such law would regulate. There were all sorts of answers, mainly participants were expecting the law to allow anyone, without any discrimination and regardless of sexual orientation or gender to be allowed to receive MAR services; to control the prices of these services; to determine psychologically and physically appropriate persons to get such services; to offer safety and protect the rights of MAR users.

*“I’m specifically talking about one person. I don’t need to mention if this person is heterosexual, female, male, intersex, transsexual or whoever has the problem with those things the services should be offered to them. Without judgment or discrimination.”* (KOS 02, 20 years, male, heterosexual, Muslim)

*“…regulate the super-limited services that aren’t offered in public hospitals of Kosovo. Have a control of prices and not leave it to will of “businessman” that opened the hospital.”* (KOS 04, 30 years, transgender female to male, homosexual, Muslim)

*“A law that frames these procedures, to see who is able psychologically and physically to go through.”* (KOS 08, 25 years, female, heterosexual, Muslim)

Access to information issue related to MAR was evident, with participants stating their thoughts that they have not come across too much, if any, publicity and information about MAR in Kosovo’s environment. As a possible sources and channels for getting the information, many have indicated health institutions and health professionals, as well as internet as a possible source of information, but at the same time some of them have pointed out that internet sources are not in local language. We have also asked them about the desired content and channel of provision of such information and we have received various answers. Many of them pointed out they were interested in success rates and safety of MAR techniques, what procedures are available, the technical aspects of different techniques and legal issues regarding MAR. As for the channels, many have suggested a central web based portal that would contain as much information as possible, but also more traditional channels, like brochures, posters, dedicated TV or radio shows, as well as education of younger generations at school.

*“There is not enough publicity. As I said earlier this is a topic that is not really discussed and I think the taboo related to this topic should be there anymore. I think doctors should do TV appearances and explain to people how these IVF methods. How successful it is?”* (KOS 09, 20 years, male, heterosexual, Muslim)

*“What are the procedures that can be received in Kosovo? I would like to know what treatments they offer, which techniques are successful and which are not successful. The difference between them.”* (KOS 08, 25 years, female, heterosexual, Muslim)

*“I would like to see the websites of these clinics, their locations and every service they offer listed there. The best way would be to have a website where everything could be explained. You have the lists of doctors is there. The lists of services and treatments is there.”* (KOS 02, 20 years, male, heterosexual, Muslim)

## Discussion

The present study provides insight into current attitudes towards parenthood, knowledge about (in)fertility issues among young people in Kosovo. Our study is the first study carried out in Kosovo and the region on exploring views with young people on expectations and representation of parenthood, procreation and infertility compared with other European countries which had several strengths. First, heterogeneity of young participants in the whole research study with high participation in order to gain various insights about (in)fertility and parenthood. Second, it was used originally developed interview guide and validated by the researchers. Final, there are no other studies where traditional gender patterns still prevail and demonstrated taboos and stigmatization on (in)fertility and specific cultural and traditional norms are linked to parenthood and reproduction.

By making explicit connection with all three emerged themes, the complex ways by which care for (in)fertility are present become apparent. Our findings show that majority of respondents, both male and female, expressed a desire to become parents and generally had good attitudes on conceptions of parenthood.

An important aspect of traditional norms that is highlighted in our findings is gender aspect in parenting. Most people consider parenthood to be an essential aspect of life ( Daniluk, 2001). Parenthood arises as a component of the social structure formed by the institution of marriage; this structure is heavily impacted by traditional lifestyles, resulting in the formation of distinct roles ( Gurkan, 2021). Having children was more essential to women than it was to males. Men were less likely to think they would seek MAR treatment and more likely to think they would forgo having children if faced with infertility. Though they all acknowledge that there is strong social pressure to become a parent, many of them also believe that it should be up to each individual whether or not they choose to become parents. Some refer to being a parent as a responsibility, a dream, an accomplishment, a holy blessing, a bodily and emotional need, or a gratifying experience. However, generally, the results about parenthood are increasingly viewed as an obligation for both men and women rather than a choice. There, the concept of "parenthood as choice" and traditional gender norms are less at odds ( Struyf *et al.*, 2023).

Our findings contribute to several examples to ongoing discussions in the literature on parenthood, (in)fertility and MAR aspects. The findings from our interviews demonstrated that parenthood is still gender related ( Gurkan, 2021; Paschal *et al.*, 2011). In our study, some participants perceived that bigger burden falls to mothers because they consider that the *essentialisation of motherhood* which is imposed by traditional gender roles and pro-natalistic important policies ( Tarkhanova, 2018).

Other young participants, however, seemed to have expectations that father’s role is to provide financial support or is considered as a breadwinner in general. This seemed particularly true for most young participants in the study sample, suggesting how best to explore the knowledge about parenthood patterns and roles in Kosovo.

Our findings also contribute to the current research discussion on (in)fertility importance among young people and how it can affect their life ( Boivin *et al.*, 2007; Facchin *et al.*, 2020). The interviewed participants in the study, considered male (in)fertility as a taboo in their social networks, not much discussed in public. The communication difficulties among young people and their peers that we have identified are examples how implicit knowledge about infertility can influence their behaviour and quality of life ( Schick *et al.*, 2016). For example, the dominant belief among majority of the participants is that woman is judged, blamed a priori for being infertile, actually takes the biggest pressure from the environment. This showcase strengthens the understanding of stigmatization of (in)fertility between young people and revealed differences in knowledge about childness and causes of being infertile. This statement supports observations from Vialle *et al.* (2023) that childlessness in general is viewed as a social pressure and is not desired particularly in rural areas in Kosovo.

A substantial amount of lack of knowledge was found in relation to MAR techniques, highlighting the importance of detailed information about the whole process of MAR treatments. A total of 5 private ART clinics are currently based in Kosovo. Information provided on their websites was aimed toward implicitly heterosexual couples and conventional family models experiencing infertility; there was no mention of LGTB individuals or single women whatsoever (they are not permitted to access). Women were given greater attention than men, there is no any information regarding requirements for access, such as age restrictions or how to obtain access. The accounts revealed that interviewed participants were fully aware of existence of MAR techniques, however, the details and type of right synthesized information are deficient to avoid further confusion with people. Although, we did identify various gaps in understanding the MAR process discussed above, it is the health authorities along with MAR clinic that provide suitable information and thus it is essential that the persons who are seeking fertility treatment should be informed of available alternatives to MAR. Barriers identified in our research that have been known to influence access to MAR clinics, but have less frequently been studied in the context of fertility, include finance and economic reasons as most of the clinics are private. Majority of participants have stressed that the biggest problem in accessing such services is financial incapacity of the population, as these services are not covered and should be paid out of pocket. Our study serves as an example how private MAR clinics decrease the access to people who want to conceive with alternative methods because of the increased costs.

## Conclusion

This study is the first of its kind in the South-East Europe and its results are useful for domestic healthcare stakeholders and policy-makers. The majority of male and female respondents wished to have children and they had an overall positive attitude towards perceptions of parenthood, but contingent upon having a favourable socio-economic situation. Perceptions about infertility have a clear gender component, being considered a problem that particularly affects women and revealing taboos associated with female infertility. Our findings indicate that there is a clear gap of knowledge and relevant need for information about (in)fertility, MAR methods to the young people in Kosovo. In our views, it is recommended that health authorities create a catalogue of informed consent forms and information sheets that comply with current regulations in Kosovo. Persons seeking fertility treatment should be informed of available alternatives to MAR, including adoption and the possibility of treating infertility through biomedical intervention.

## Limitations

The study participants were mainly from Prishtina, the capital city of Kosovo, a few came from other regions. The capital city is the biggest and most populated city in Kosovo where many young people migrate for education. Young people from other cities may have different results due to cross-cultural differences. 

## Ethical approval and consent to participate

This project (n°2021.004) received ethical approval from the Research Ethics Committee of the University of Navarra on 29 January 2021. The Research Ethics Committee reviewed the Protocol, Patient Information sheet and Informed Consent dated on 26/01/2021. On 29/02/2021 the Committee issued a statement for the realization of the project since it has considered that if conforms to the essential ethical principles and with the deontological principles of the Institutions.

All interview participants provided informed consent. For in-person interviews, consent forms were signed physically, while for online interviews, the forms were sent as PDFs and returned signed by participants. The consent form indicated that transcripts would be used for preparing scientific articles and reports related to B2-InF. Additionally, participants provided audio-recorded consent at the beginning of each interview.

## Data Availability

The data underlying these results cannot be shared due to confidentiality concerns. The sensitive nature of the information poses a risk of exploitation by the fertility industry for commercial purposes. To address these concerns, the Grant Agreement (nr. 872706 — B2-InF) signed between the Research Executive Agency of the European Commission and all B2-InF project partners specifies that the dissemination level of the interviews, as well as the corresponding reports and thematic analyses, is classified as "confidential." This designation is explicitly defined as "accessible only to members of the consortium (including the Commission Services)" To request access, please email the corresponding author. Access will be granted after verifying that the data will be used exclusively for scientific research purposes and that all researchers involved in the study have no conflicts of interest.

## Software availability statement

The qualitative analysis in this article was performed using Atlas.ti (version 23.2.1), a proprietary qualitative data analysis software. For more information about Atlas.ti, visit the Atlas.ti website www.atlasti.com. For those without access, equivalent analyses can be performed using open-source alternatives such as Taguette ( https://www.taguette.org/).

## References

[ref-1] Archer NP Langlois PH Suarez L : Association of paternal age with prevalence of selected birth defects. *Birth Defects Res A Clin Mol Teratol.* 2007;79(1):27–34. 10.1002/bdra.20316 17120236

[ref-2] ATLAS.ti Scientific Software Development GmbH: ATLAS.ti Mac (version 23.2.1). [Qualitative data analysis software],2023. Reference Source

[ref-30] Boivin J Bunting L Collins JA : International estimates of infertility prevalence and treatment-seeking: potential need and demand for infertility medical care. *Hum Reprod.* 2007;22(6):1506–1512. 10.1093/humrep/dem046 17376819

[ref-3] Brunborg H : Report on the size and ethnic composition of the population of Kosovo. Case of Slobodan Milosevic (IT-02-54), ICTY, La Haye,2002. Reference Source

[ref-4] Čipin I Zeman K Međimurec P : Cohort fertility, parity progression, and family size in former Yugoslav countries. *Comparative Population Studies.* 2020;45. 10.12765/CPoS-2020-18

[ref-5] Cleary-Goldman J Malone FD Vidaver J : Impact of maternal age on obstetric outcome. *Obstet Gynecol.* 2005;105(5 Pt 1):983–990. 10.1097/01.AOG.0000158118.75532.51 15863534

[ref-6] Daniluk JC : Reconstructing their lives: a longitudinal, qualitative analysis of the transition to biological childlessness for infertile couples. *J Couns Dev.* 2001;79(4):439–449. 10.1002/j.1556-6676.2001.tb01991.x

[ref-7] Facchin F Leone D Tamanza G : Working with infertile couples seeking assisted reproduction: an interpretative phenomenological study with infertility care providers. *Front Psychol.* 2020;11: 586873. 10.3389/fpsyg.2020.586873 33391106 PMC7773748

[ref-8] Frejka T Gietel-Basten S : Fertility and family policies in Central and Eastern Europe after 1990. *Comparative Population Studies.* 2016;41(1):3–56. 10.12765/CPoS-2016-03en

[ref-9] Gurkan T : A qualitative study on the perception of fatherhood. *Eur J Educ Sci.* 2021;8(2):42–59. 10.19044/ejes.v8no2a42

[ref-10] Kaser K : Patriarchy after patriarchy: gender relations in Turkey and in the Balkans, 1500–2000. Lit Verlag,2008. Reference Source

[ref-11] Krasniqi M : The role of tradition in self-reproductive knowledge among Albanians. *Albanological Traces - Folklore and Ethnology.* 1990;19.

[ref-12] Latifi T : Family, kinship relations and social security in Kosovo since the war of 1999. [Doctoral dissertation, Graz],1999. Reference Source

[ref-14] Lerch M : Fertility and union formation during crisis and societal consolidation in the Western Balkans. *Popul Stud (Camb).* 2018;72(2):217–234. 10.1080/00324728.2017.1412492 29357746

[ref-15] Paschal AM Lewis-Moss RK Hsiao T : Perceived fatherhood roles and parenting behaviors among African American teen fathers. *J Adolesc Res.* 2011;26(1):61–83. 10.1177/0743558410384733

[ref-16] Predojević J : Fertility transition in works of Albanian authors from Kosovo and Metohia. *Stanovnistvo.* 2001;39(1–4):131–156. 10.2298/STNV0104131P

[ref-17] Pushka A : Factors of birth rate and their quantification. *Problems of population renewal policy in Serbia.* 1989.

[ref-18] Regushevskaya E Hemminki E Klemetti R : Postponing births - Comparing reasons among women in St Petersburg, Estonia, and Finland. *Finnish Yearbook of Population Research, XLVIII.* 2013;127–145. Reference Source

[ref-19] Sartorius GA Nieschlag E : Paternal age and reproduction. *Hum Reprod Update.* 2010;16(1):65–79. 10.1093/humupd/dmp027 19696093

[ref-13] Schick M Rösner S Toth B : Effects of medical causes, role concepts and treatment stages on quality of life in involuntary childless men. *Andrologia.* 2016;48(9):937–942. 10.1111/and.12519 27739143

[ref-20] Schmidt L Sobotka T Bentzen JG : Demographic and medical consequences of the postponement of parenthood. *Hum Reprod Update.* 2012;18(1):29–43. 10.1093/humupd/dmr040 21989171

[ref-21] Shiffman J Skrabalo M Subotic J : Reproductive rights and the state in Serbia and Croatia. *Soc Sci Med.* 2002;54(4):625–642. 10.1016/s0277-9536(01)00134-4 11848279

[ref-22] Struyf J Hens K Rozée V : Gender aspects in parenthood conceptions of young Europeans. A critical reflection on the European Project B2-InF [version 1; peer review: 2 approved with reservations]. *Fam Relatsh Soc.* [Forthcoming],2023. 10.12688/openreseurope.18210.1

[ref-23] Tarkhanova O : Essentializing motherhood: the Ukrainian woman in policy debates. *InterDisciplines: Journal of History and Sociology.* 2018;9(1). 10.4119/indi-1053

[ref-24] The World Bank: Fertility rate, total (births per woman) - Switzerland.2023; Retrieved October 15, 2023. Reference Source

[ref-25] Vialle M Struyf J Hens K : Between acceptance and rejection: young Europeans' ambivalent relationship with the procreative norm. *J Gend Stud.* [Forthcoming],2023.

