# Exploring MAR perceptions in Kosovo – disparities in knowledge about MAR among young people

**DOI:** 10.12688/openreseurope.18974.4

**Published:** 2026-01-23

**Authors:** Vera Dimitrievska, Jana Meloska, Virginie Rozée, Manon Vialle, Kristien Hens, Joke Struyf, Anna De Bayas Sanchez, Michaela Fuller Fuller, Francisco Güell, María López-Toribio, José Miguel Carrasco

**Affiliations:** 1 Research Department, HeatlhGrouper, Skopje, North Macedonia, Skopje, 1000, North Macedonia; 2 University of American College, Skopje, North Macedonia; 3 INED, Research Unit “Sexual and reproductive Health and Rights”, Aubervilliers, France; 4 University of Antwerpen, Department of Philosophy, Antwerpen, Belgium; 5 Medistella Mediversvm s.r.o., Management, Prague, Czech Republic; 6 Institute for Medical Humanities 1st Faculty of Medicine of Charles University, Prague, Czech Republic; 7 University of Navarra, ICS, Pamplona, Spain; 8 Universidad Anáhuac México, Facultad de Bioética, Ciudad de México, Mexico; 9 APLICA Investigación y Traslación Cooperative, Madrid, Spain

**Keywords:** Parenthood, (in)fertility, MAR techniques, perceptions, young people, Kosovo

## Abstract

Research on knowledge and perception about (in)fertility and Medically Assisted Reproduction (MAR) techniques among young people in Kosovo is lacking. Part of the European Project’s B ^2^-InF’s team conducted a qualitative study in Kosovo with young adults (18–30) in 2021 assessing perceptions and views related to MAR and information provided by the MAR clinics in Kosovo. Main objective of this study is to explore perceptions about motherhood/fatherhood and to understand (in)fertility issues among young people in Kosovo. It is focused to delve more into MAR knowledge and techniques of young people in Kosovo and in general. Fifteen interviews with young people living in Kosovo were conducted between June and July 2021. A total of 3 themes emerged during the qualitative analysis and were grouped in three main theme levels. The first level, perceptions about motherhood and fatherhood among young people in Kosovo is about different views of young people, desires about becoming parents, and what is their personal attitude towards parenting. The second, analyzing how (in)fertility is perceived by young people from a gendered perspective. The third, MAR perceptions and new techniques that are prevailing in the global sense as different access opportunities are present. The findings from our interviews demonstrated that parenthood is still gender related. Male (in)fertility is often considered a taboo among the young people in Kosovo. A substantial amount of lack of knowledge was found in relation to MAR techniques, highlighting the importance of detailed information about the whole process of MAR treatments.

## Background

Over the past decades the research about parenthood/motherhood, fertility and treatments about infertility gained an immense attention in research and online forums. The research studies show that people view being a parent as an essential aspect of their life cycle ( Daniluk, 2001). After seeing a substantial decline of fertility rate levels in entire Europe in the last decades also the parental age started to increase ( Regushevskaya *et al.*, 2013; Schmidt *et al.*, 2012). This trend affected also the countries from Western Balkan during the 1990s in a way that fewer children are born per couple in different age from different reasons. The main reasons are cultural and economic factors. From one point of view, there have been deep-rooted traditions, the predominance of conservative family values within a predominant Muslim population, a low acceptance of birth control and modern contraception, high marriage rates, and low economic development. From another point of view, there is an unclear and generally constant pessimistic political situation, persistent agrarian settlement, poor female education, and low female economic activity ( Čipin *et al.*, 2020; Frejka & Gietel-Basten, 2016)

Numerous studies have shown that increasing parental and maternal age is associated with prolonged gestation and elevated risks during pregnancy, including miscarriage ( Archer *et al.*, 2007; Cleary-Goldman *et al.*, 2005; Sartorius & Nieschlag, 2010).

The (in)fertility patterns affected the Western Balkan countries in the late 90s. Demographic structure of fertile rates levels among women in this region has changed drastically. While in 1991 the average number of children per woman in Kosovo was 3.25 ( Brunborg, 2002), by 2019 this figure had declined to 1.55 ( World Bank, 2023)—a rate that remains higher than the European average but reflects a rapid demographic transition. The average mother's age for her first childbirth this year was 29 ( World Bank, 2023). Postponing family formation compared to Western Europe countries is slowly declining especially in Kosovo due to influence of various factors such as cultural, traditional, political and ideational aspects ( Lerch, 2018). Literature argues that the societal backdrop was a significant factor in this fertility behaviour. The influence of religious discourses and government measures of pro-natal policies place an extensive emphasize on women’s role and traditional values in upbringing the child ( Shiffman *et al.*, 2002). Moreover, Kosovo’s secular tradition of customary law and its cohesive social systems have played a significant role in shaping and sustaining traditional relational structures. ( Kaser, 2008). In certain places, tradition and religion are still combined to form a single entity. Although their objectives are slightly different, tradition promotes as many families as possible since it is a symbol of its power, while the religious values have as many members as possible ( Krasniqi, 1990).

A few studies looked at the gender aspects and fertility issue in Kosovo ( Predojević, 2001; Pushka, 1989). Women's inferior status within the family and society was largely determined by their lack of agency in decision-making and their acceptance of the necessity of their current relationships. According to Predojević (2001) a woman in rural areas still obeys to her husband's will and has little options to further her lower-level education. She was forced to choose between total subjugation and the inability to alter his standing in the community, trapping herself in an endless cycle. She thereby assumes the role of "guardian of tradition," ensuring the continuation of the patriarchal lifestyle despite the fact that it is based on principles that are turned against women ( Latifi, 1999; Pushka,1989).

Research body on women’s reproductive roles in Kosovo has long relied on late- and post-socialist literature, which effectively documented the importance of patriarchal norms and extended family structures. However, continued dependence on these older frameworks’ risks depicting gender relations as static. It overlooks the profound socio-demographic transformations seen across the Western Balkans over the last two decades. 

Latest literature converges with and complicates the existing scholars on Western Balkan pronatalism. While scholars like Vasić (2021) and Kontogiannis (2024) highlight the persistence of traditional family values across the region, our data from Kosovo suggests a unique contextual specificity: a 'negotiated' form of patriarchy where youth do not blindly follow tradition but pragmatically adapt it to post-war socioeconomic precariousness. Unlike neighboring post-socialist contexts where the state has partially re-entered the welfare space, the near-total retreat of the state in Kosovo has forced a reliance on the extended family that re-solidifies patrilineal power, even among youth who hold modern educational aspirations. By situating Kosovo within this comparative regional context, this study captures the tension between historical continuity and contemporary change without resorting to essentialized representations ( Apostolovska Toshevska *et al.*, 2024).

Micro-level evidence from neighboring contexts illustrates how reproductive attitudes among Albanian women are shaped by the interplay of persistent normative expectations and shifting socioeconomic realities. In their study of the Arachinovo Municipality in North Macedonia, ( Apostolovska Toshevska *et al.*, 2024) demonstrate that while marriage and motherhood remain central values, women’s reproductive choices are increasingly contingent upon education, employment, and economic security. Notably, the study identifies a generational divide: younger, more educated women exhibit greater ambivalence toward early or high fertility, even as they navigate social pressures to conform. These findings suggest that patriarchal norms within Albanian-speaking communities in the Western Balkans are not static; rather, they are actively negotiated within specific structural constraints—a dynamic that is highly relevant for interpreting contemporary behaviors in Kosovo.

Comparative research from other Central and Eastern European contexts has shown that strong pronatalist norms and traditional gender expectations continue to influence reproductive decision-making and the social framing of infertility. For instance, Szalma (2021) argues that in pronatalist societies, assisted reproduction is perceived through the lens of gendered expectations about motherhood and male virility, with male infertility often remaining a social taboo. Integrating this broader perspective allows the current study to position the Kosovar context within a wider regional discourse on how post-socialist societies negotiate modern reproductive technologies while maintaining traditional family ideals. Despite the steady fertility rates in Kosovo and all reforms, changes have embarked in the recent years. Medically Assisted Reproduction (MAR) industry is also present in Kosovo. It counts 5 private MAR clinics, highly focused on describing step-by-step procedures and medical indications of treatments. MAR techniques have been legally regulated in 2006 based on the societal context, including the clinical and biological procedures.

MAR in Kosovo is governed by a fragmented and evolving regulatory framework. While a draft Law on Reproductive Health and Medically Assisted Conception has been developed, it has not yet been fully adopted, and MAR services have historically been regulated through secondary legislation issued by the Ministry of Health. Until recently, access to MAR was almost exclusively provided through private healthcare facilities, with treatment costs borne entirely out of pocket by patients. This reliance on private provision has made access to MAR strongly dependent on financial resources, limiting availability for lower-income groups and reinforcing socioeconomic inequalities in reproductive healthcare. In late 2024, publicly provided MAR services were introduced at the Gynecology Clinic of the University Clinical Center of Kosovo (UCCK), marking a significant policy development. Under this arrangement, the first two MAR treatment attempts are offered free of charge, while subsequent cycles are available at reduced fees compared to the private sector. Despite this progress, MAR is not yet comprehensively covered by public health insurance, and many individuals and couples continue to rely on private services or out-of-pocket payments. Clarifying these conditions of access and funding is essential for contextualising reproductive decision-making and the lived experiences of individuals seeking MAR in Kosovo.

Most of the information referred to in the legal framework are aimed for heterosexual couples. Surrogacy is prohibited in Kosovo as an alternative way of establishing a family. People of same-sex relationships are still legally barred from marrying and starting a family.

## Methods

The qualitative study conducted in Kosovo as part of the European project *B2-InF* targeted young adults aged 18–30 in 2021 to explore sociocultural, gender, and legal perspectives related to medically assisted reproduction (MAR). Semi-structured interviews were used, following a common interview guide developed by the research consortium. A total of 15 interviews, lasting 40–55 minutes each, were conducted and analysed by the B2-InF team. A purposive sampling strategy was employed to capture heterogeneity across gender, age, place of residence, marital/relationship status, sexual orientation, education, and religion. Participants were primarily recruited through snowball sampling and word-of-mouth referrals facilitated by the local partner organisation, Health Grouper. Initial contacts were identified via professional and community networks, and participants were invited to recommend other eligible individuals. While most interviewees were based in Prishtina, the capital city, several participants were recruited from other areas outside the capital, allowing for limited geographic variation. We acknowledge that this recruitment approach may have favoured individuals with higher education or greater urban engagement, and that the relatively small sample size limits generalisability. These clarifications have been added to the Methods section to improve transparency and to contextualise the scope and potential limitations of the study. A standardized thematic interview guide was used and transcribed by local team members in the local language. The guide explored four main domains: (1) representations of family and parenthood; (2) perceptions and social meanings of infertility; (3) knowledge, expectations, and aspirations regarding medically assisted reproduction (MAR); and (4) desired information and perceived accessibility of MAR services. This structure enabled comparative insights into how young adults across diverse sociocultural contexts engage with reproductive futures and technologies. In appendix is a concise summary of the interview guide:

Primary criteria to select participants were heterogeneous young people from 18–30 years of age ( Table 1). The age range of 18–30 was intentionally selected to align with the core objective of the B2-InF project: to explore how young, childless individuals—those who have not yet actively considered parenthood or encountered fertility challenges—perceive assisted reproductive technologies. This group, referred to as the “not-patient” population, is of particular interest because its members are not yet shaped by personal experiences of infertility, which could significantly influence attitudes toward MAR. Moreover, this demographic is considered a key indicator of future societal norms around reproduction, family, and parenting. By focusing on this cohort, the study aims to understand whether traditional reproductive expectations persist or whether new imaginaries are emerging.

**Table 1.** Demographic characteristics of participants.

	Participants (N=15)	
	N	%
**Age**		
18–22	8	53
23–26	6	40
27–30	1	7
**Gender**		
Male	8	53
Female	6	40
Transgender female to male	1	7
**Sexual orientation**		
Heterosexual	13	86
Homosexual	1	7
Bisexual	1	7
**Occupation**		
Student	2	14
Employed	8	53
Unemployed	5	33
**Education**		
Secondary school	1	7
High school	9	60
Bachelor degree	5	33
**Residence**		
Urban area	8	53
Semi-urban area	3	20
Rural area	4	27
**Relationship**		
Yes	8	53
No	7	47
**Marital status**		
Single	10	67
Married	5	33
**Religious**		
Christian	1	7
Jewish	0	0
Muslim	13	86
Others	1	7

The study used purposive sampling technique in order to capture heterogeneity of young participants (gender, age, residence, marital status/relationship, sexual orientation, education and religion). Majority of the participants in the interviews were recruited through snowballing method and word of mouth by Health Grouper primarily from Prishtina, the capital city of Kosovo, but some interviewees came from other areas outside the capital. A thematic analysis was performed using Atlas.ti software (version 23.2.1).

For the purpose of this study, we used an exploratory approach in order to better understand what the perceptions among young people in Kosovo are and to fill the gap in the literature about the motherhood/fatherhood trajectory. There remains a lack of knowledge about (in)fertility relevance in Kosovo and MAR perceptions among the young people. Therefore, this study provides an opportunity to gain insight into fertility behaviour and reproduction as they capture views of young people who are not parents about motherhood/fatherhood and (in)fertility. Furthermore, it also explores the knowledge and perceptions of young people about MAR techniques in Kosovo and in general.

This project (n°2021.004) received ethical approval from the Research Ethics Committee of the University of Navarra on 29 January 2021. All interview participants provided informed consent. For in-person interviews, consent forms were signed physically, while for online interviews, the forms were sent as PDFs and returned signed by participants. The consent form indicated that transcripts would be used for preparing scientific articles and reports related to B2-InF. Additionally, participants provided audio-recorded consent at the beginning of each interview.

## Analysis of data

All interviews were conducted in Albanian, audio-recorded, transcribed, and translated into English. The analysis followed a hybrid deductive-inductive approach. Initially, a preliminary set of pre-defined codes—reflecting the research consortium’s thematic interests—was developed. Concurrently, the local researcher created an initial code structure inductively from a subset of transcripts to capture emergent perspectives. Using Atlas.ti software, the researcher independently applied this final code structure to all transcripts. Discrepancies were discussed with consortium members, and consensus was achieved through a negotiated process. To enhance credibility, the emergent themes were also presented to participants for validation. Following coding, codes were aggregated into subthemes and higher-order themes, reflecting increasing levels of abstraction. Three overarching themes emerged: (1) *Perceptions of motherhood and fatherhood*, capturing young people’s desires, attitudes, and views on parenting; (2) *Relevance and importance of fertility*, exploring the significance of (in)fertility from gendered perspectives; and (3) *MAR perceptions and access to new reproductive technologies*, highlighting awareness of available techniques and perceived opportunities.

## Results

### Perceptions about motherhood and fatherhood among young people in Kosovo

The theme fatherhood/motherhood perceptions among young people in Kosovo is linked to planning, differentiation, and desire to become parents. These issues were key across the interviews. Participants conceptualize the transition to parenthood as a milestone reserved for 'fully grown persons'—a status defined not merely by age, but by the achievement of socioeconomic stability. With most of the participants we observed that parenthood is seen as a primary role or something that is a priority in their life. Desire to become parents was present across the interviews with strong positive attitudes towards parenthood and motherhood. Perceptions about planned parenthood are considered as a responsible feature for a future parent, which is linked to the belief that it is very important to be mature enough before becoming a parent.

*“Parenthood is a big responsibility but is a right that can’t be denied to no one. So everyone that considers themselves to be ready to offer the love, care and take the responsibility to be a parent then no one can deny the right to anyone to do that.”* (KOS 04, 30 years, transgender female to male, homosexual, Muslim)

Participants also attributed a concern to irresponsible parenting especially among parents at young age like 17–22 years old, since people in Kosovo become parents at young age. Nevertheless, they do believe that unplanned pregnancy may become a basis for certain mental health problems like depression or anxiety. On the same note, participants stated that being financially secured is an important aspect of raising children but also on the other hand it was expressed that some couples do not have access to abortion or knowledge about planning a pregnancy.

*“Because when they are not ready financially, psychologically and physically, if so I think it’s better not to have children.”* (KOS 08, 25 years, female, heterosexual, Muslim)

A highly prominent feature emphasized by the participants was differentiation among motherhood vs. fatherhood. There appeared to be a difference in perceptions about parenthood and participants belief that bigger burden falls to mothers/females, because they consider that the mothers have a stronger connection with the children as a result of the linkage with the psychological process during pregnancy, birth/delivery, breastfeeding and the hormonal changes.

*“The child's connection with the mother is stronger than with the father, not that with the father it is not a strong connection but with the mother it is much stronger in my opinion and at the same time the mother's connection with the child is stronger because she keeps it for 9 months in the womb.”* (KOS 14, 21 years, male, heterosexual, Muslim)

In relation to parenthood, participants believe that mother’s role is more demanding in the whole process of taking care of the children. Mothers carry a burden to raise the child in the intensive motherhood according to participants, which is also linked to care, education and organizing the household, because they believe that women are more capable for it than men. Participants reflected that men are expected to provide financial support or are considered a breadwinner in general.

*“For fathers, parenthood is seen as something that they should provide financially and for mothers it has to do a lot more with the education aspect of the children.”* (KOS 03, 20 years, male, heterosexual, None)

Social pressure to become parents plays considerable role among young people in Kosovo. Participants felt more controlled by the society to be a parent, as it is relevant for their social setting. Although many participants acknowledge that parenting is an individual choice, there is a social pressure to become parent. A few participants stated that they do not choose consciously to have children, but they are led by the expectations from their older generations. Related to this, they reported obligation to be a parent is perceived in various ways, like to care for the child as duty, from religious view it is something that child would bring into the life and increase the birth rate in the country.

*“Because exactly from the social pressure they end up being a parent to continue the cycle of life and descendants. They don’t really feel they want to become a parent or are really ready psychologically or emotionally to be one.”* (KOS 03, 20 years, male, heterosexual, None)

*“For Muslim people I believe it has a lot of importance because everyone that is a Muslim want to have descendants and it is really important for our community and Muslims to have kids and continue the religion and everything.”* (KOS 05, 21 years, female, heterosexual, Muslim)

Participants have shared their opinions that society expects everyone to have children as that is perceived as a norm, and not having children can be seen as a form of a taboo. This pressure is even more emphasised in women, as women are perceived as a means of reproduction. Majority of participants locate the pressure for having children in the religion.

*“The traditional lifestyle becomes so powerful that people don’t decide things, but just do them routinely”. (KOS 09,* 26 years, female, heterosexual, Muslim *)*

By interrogating these views, we see that the perceived 'necessity' of children is embedded in power relations that prioritize patrilineal continuity. Consequently, the ambivalence expressed by some youth regarding MAR represents a negotiation with a moral economy of reproduction that remains skeptical of technological interventions that might disrupt traditional concepts of kinship and biological 'purity' ( Cicerchia, 2023).

### Relevance/Importance of (in)fertility in Kosovo environment

Interviews show that young people understand the meaning of (in)fertility and perceive it as an important issue, as most of them believe they are fertile and hope to have children of their own. However, the topics like sexuality, fertility, women’s body are less discussed, while both male and female participants claimed that being infertile is a curse from God, especially for women. Therefore, women who are childless feel more societal pressure and judgement by close circle of people even further, regardless if she is voluntarily or involuntarily childless. General perception from the young people in Kosovo is that women are described as ‘reproductive machines’. Participants don't think a woman's capacity to conceive will change after she reaches 30; rather, they view fertility as a gift as long as a woman is capable of bearing children. Several participants have emphasized that women are more frequently affected with infertility compared to males.

*“Being fertile is one of the most important things, me as a woman that wants to have three or four children, depends on how God will bless me, for me it is really important to be fertile and not to have to use other alternative methods to create a family.”* (KOS 09, 26 years, female, heterosexual, Muslim)

*“I think most of the times females are the ones who have the most problems with infertility, those who have problems with the uterus as much as I have heard or read. It is the female who has the most difficulty.”* (KOS 02, 20 years, male, heterosexual, Muslim)

Male (in)fertility is often considered a taboo among the young people in Kosovo and not much discussed in public. Since this topic is hardly discussed, then understanding is lacking, particularly in rural regions, in addition to a few participants expressing extreme viewpoints and misunderstanding about male infertility. However, they are expected to procreate and carry on the family name, so men are also believed to experience infertility. Sometimes a man would prefer to undergo and seek a divorce and marry another woman than to get fertility test. Men who are infertile are perceived as being weaker and having lost their male ego.

*“Men […. ] add more people to the family, men carry this responsibility in our society”* (KOS 03, 20 years, male, heterosexual, None).

Even if they are infertile, men are expected to hide their vulnerability and sensitivity, since they are not allowed to be infertile. Because of the male infertility being taboo, women are consistently held accountable of their infertility.

Many participants agreed that infertility is increasing, with both men and women experiencing difficulties, albeit in different ways. Women who are infertile, according to the participants are not able to successfully realize their role as a mother. Moreover, women are more emotional and sensitive than men with a strong desire to create a family. As for the possible causes of infertility, participants have pointed out various possible causes. Many of them agreed that age is an important factor for infertility, and various intrinsic and extrinsic factors have been mentioned by two participants, such as stress, unhealthy lifestyles, genetics, anatomy of female genital organs, genital infections, environmental and air pollution. Knowledge about potential reasons for (in)fertility are missing.

*“For me it is really important to have children then of course infertility would not let me accomplish my need and want to have children that I feel.”* (KOS 08, 25 years, female, heterosexual, Muslim)

*“For a person to be fertile they must first have a healthy life and not have too much stress in life, and I strongly believe that the majority of the population is fertile, but this also depends on environmental pollution, air pollution, or how people live their life, it also depends genetically.”* (KOS 13, 26 years, female, heterosexual, Muslim)

The taboo surrounding male infertility observed in our data echoes broader findings on how pronatalist discourses reinforce silence around men’s reproductive vulnerability. In the Kosovar context, infertility is perceived as a threat to masculine identity—a 'loss of ego'—leading to the social invisibility of male infertility and disproportionate blame placed on women. This social logic reveals that reproductive success remains closely tied to social status and normative expectations of masculinity, where men are expected to hide vulnerability to maintain their position within the gendered power structure ( Sahoo *et al.*, 2025)

### Medically Assisted Reproduction (MAR) perceptions – what people think about it

We discussed general knowledge and personal perceptions about MAR with the participants. Overall, participants were not very familiar with the techniques available in general and those available in Kosovo, but most of them knew or at least have heard about In Vitro Fertilization (IVF). Several participants could identify some other techniques as well, but many of them acknowledged that the procedures and the process for MAR techniques are not known, and they practically don’t know anything about it. As far as success rates were concerned, most of participants believe MAR techniques are pretty much successful. When asked whether they would undergo some sort of MAR intervention, in case it was needed, we received heterogeneous responses. One stated they would undoubtedly undergo whatever intervention is needed, while others were more reserved and replied they would prefer some more conservative treatments or an adoption as a method. Their main concerns with MAR were lack of information and anxiety about them, concerns over possible defects or syndromes or transmission of infections to the baby.

*“No, I do not have information about these services. I, as 99% of the population of Kosovo, am not informed about these things.”* (KOS 07, 21 years, male, heterosexual, Muslim)

*“… No way for artificial methods, or with sperm donation. If there is any treatment that makes me or my partner fertile, depending on who is infertile, then YES. Otherwise, adoption would be the option.”* (KOS 14, 21 years, male, heterosexual, Muslim)

*“Yes, if I would want to have a kid and I wouldn’t be able to have one then without a doubt I would try these techniques.”* (KOS 05, 21 years, female, heterosexual, Muslim)

*“If something goes wrong the child can be born with defects, this is my only concern. That the child could have defects or the child could be born with any kind of syndrome.”* (KOS 09, 26 years, female, heterosexual, Muslim).

In the interviews we wanted to hear participants’ views about the accessibility of the MAR clinics. Some disagreeing opinions were brought out, as some believe that MAR may pose significant financial burden to potential clients, while others say that these interventions are practically free. Some participants also believe candidates for MAR should be initially assessed and fulfil certain requirements. Majority of participants have stressed that the biggest problem in accessing such services is financial incapacity of the population, as these services are not covered by the health insurance and should be paid out of pocket. Also, they have pointed out that these services are offered in limited number of clinics and that there is generally low confidence in health services provided in Kosovo. They have pointed out the low awareness in general public about the issue of MAR as an additional factor adversely influencing the accessibility of these services. While participants frequently cited the extended family as a crucial source of emotional and financial support for MAR, this reliance should be interrogated as an extension of the patrilineal gender regime. In the absence of state-funded reproductive healthcare, the family becomes a moral economy where financial assistance is often contingent upon the reproduction of traditional kinship lines ( Cicerchia, 2023). This 'support' is thus not neutral; it functions as a power relation that reinforces the surveillance of women’s bodies by the kin group, ensuring that reproductive technologies are used to fulfill collective expectations of lineage rather than individual autonomy.

*“I think people with good financial income have the easiest access to these techniques and families that have knowledge related to these techniques.”* (KOS 08, 25 years, female, heterosexual, Muslim)

*“In Kosovo there is a lack of clinics and places that offer these opportunities.”* (KOS 11, 22 years, female, bisexual, Muslim)

*“I think it is not easy because most hesitate to ask and search for these kinds of treatments and services because it is normalized. Second the fact that there is no health insurance it makes it unaffordable for the most and third and most important as I mentioned first that for someone to look up for a service this service should be more talked about by the society.”* (KOS 04, 30 years, transgender female to male, homosexual, Muslim)

The participants’ preoccupation with the high cost of MAR reflects a deeper social logic characteristic of post-socialist contexts: the commodification of reproduction. When young people describe MAR as 'unreachable,' they are articulating the structural inequalities of a health system where reproductive rights are determined by market access. This creates a moral economy where only the economically secure are 'permitted' to overcome infertility, reinforcing a system of stratified reproduction that shapes who is allowed to participate in the 'normative' experience of parenthood in contemporary Kosovo.

*“I’m specifically talking about one person. I don’t need to mention if this person is heterosexual, female, male, intersex, transsexual or whoever has the problem with those things the services should be offered to them. Without judgment or discrimination.”* (KOS 02, 20 years, male, heterosexual, Muslim)

*“…regulate the super-limited services that aren’t offered in public hospitals of Kosovo. Have a control of prices and not leave it to will of “businessman” that opened the hospital.”* (KOS 04, 30 years, transgender female to male, homosexual, Muslim)

*“A law that frames these procedures, to see who is able psychologically and physically to go through.”* (KOS 08, 25 years, female, heterosexual, Muslim)

Access to information issues related to MAR was evident, with participants stating their thoughts that they have not come across too much, if any, publicity and information about MAR in Kosovo’s environment. As possible sources and channels for getting the information, many have indicated health institutions and health professionals, as well as internet as a possible source of information, but at the same time some of them have pointed out that internet sources are not in local language. We have also asked them about the desired content and channel of provision of such information and we have received various answers. Many of them pointed out they were interested in success rates and safety of MAR techniques, what procedures are available, the technical aspects of different techniques and legal issues regarding MAR. As for the channels, many have suggested a central web based portal that would contain as much information as possible, but also more traditional channels, like brochures, posters, dedicated TV or radio shows, as well as education of younger generations at school.

*“There is not enough publicity. As I said earlier this is a topic that is not really discussed and I think the taboo related to this topic should be there anymore. I think doctors should do TV appearances and explain to people how these IVF methods. How successful it is?”* (KOS 09, 20 years, male, heterosexual, Muslim)

*“What are the procedures that can be received in Kosovo? I would like to know what treatments they offer, which techniques are successful and which are not successful. The difference between them.”* (KOS 08, 25 years, female, heterosexual, Muslim)

*“I would like to see the websites of these clinics, their locations and every service they offer listed there. The best way would be to have a website where everything could be explained. You have the lists of doctors is there. The lists of services and treatments is there.”* (KOS 02, 20 years, male, heterosexual, Muslim)

## Discussion

This study provides insight into current attitudes towards parenthood, knowledge about (in)fertility issues among young people in Kosovo. It is the first study carried out in Kosovo and the region on exploring views with young people on expectations and representation of parenthood, procreation and infertility compared with other European countries which had several strengths. Firstly, heterogeneity of young participants in the whole research study with high participation in order to gain various insights about (in)fertility and parenthood. Secondly, it used originally developed interview guide validated by the researchers. Finally, there are no other studies where traditional gender patterns still prevail and demonstrated taboos and stigmatization on (in)fertility and specific cultural and traditional norms are linked to parenthood and reproduction. 

By making explicit connection with all three emerged themes, the complex ways in which care for (in)fertility are presented become apparent. Our findings show that majority of respondents, both male and female, expressed a desire to become parents and generally had good attitudes on conceptions of parenthood.

The findings from this study resonate with comparative analyses from other Central and Eastern European contexts, where pronatalist norms continue to reinforce essentialized gender roles in reproduction. Szalma (2021) demonstrates that in such societies, women are often seen as primary bearers of reproductive responsibility, while motherhood is morally idealized as a social duty. This perspective reflectes the attitudes expressed by many young participants in our study, who viewed motherhood as both a natural and socially expected role. The persistence of these norms in Kosovo suggests that fertility and parenting remain deeply embedded in a pronatalist moral order that shapes individual aspirations and perceptions of reproductive responsibility. Participants’ emphasis on the 'naturalness' of parenthood should not be viewed merely as a personal preference, but as an expression of the prevailing gender regime in post-socialist Kosovo. Within this regime, women’s social citizenship is often tethered to their reproductive capacity ( Apostolovska Toshevska *et al.* (2024)

The observed disparities in MAR knowledge based on religious affiliation should not be interpreted as a result of religion acting as a static or monolithic barrier. Instead, religious identity functions as a socially constructed lens through which young people in Kosovo filter and evaluate bioethical information. Our findings suggest that religious frameworks are internally heterogeneous; individuals within the same faith community may prioritize different aspects of 'tradition'—such as the sanctity of biological lineage versus the ethical imperative to seek medical healing. By viewing religiosity as a negotiated framework, we can see that knowledge levels are historically contingent, shaped by the specific interaction between inherited values and the modern structural reality of healthcare access in the Western Balkans. Rather than reifying tradition as an unchanging cultural prescription, this study highlights how young people actively reconcile their faith with the scientific possibilities of MAR, demonstrating that religious influence is fluid and responsive to contemporary socioeconomic shifts ( Kontogiannis, 2024).

An important aspect of traditional norms that is highlighted in our findings is gender aspect in parenting. A systematic comparison reveals that while both genders identify motherhood as more 'demanding,' the social meanings they attach to this demand differ significantly. Male participants often framed the 'biological and emotional bond' of motherhood as an essentialized ( Daniluk, 2001), almost sacred duty—a view that serves to justify the secondary role of fathers in domestic spheres. Conversely, female respondents articulated this 'demand' not as a natural bond, but as a structural burden and a source of potential professional 'sacrifice.' By moving beyond descriptive summaries, we see that what male participants call 'nature' is interrogated by female participants as a gendered regime of labor that limits their autonomy in the post-socialist labor market

Parenthood arises as a component of social structure formed by the institution of marriage; this structure is heavily impacted by traditional lifestyles, resulting in the formation of distinct roles ( Gurkan, 2021). Almost all participants acknowledge that there is strong social pressure to become a parent, many of them also believe that it should be up to each individual whether or not they choose to become parents. Some refer to being a parent as a responsibility, a dream, an accomplishment, a holy blessing, a bodily and emotional need, or a gratifying experience. However, generally, the results about parenthood are increasingly viewed as an obligation for both men and women rather than a choice. There, the concept of "parenthood as choice" and traditional gender norms are less at odds ( Struyf *et al.*, 2023).

Our findings contribute to several examples to ongoing discussions in literature on parenthood, (in)fertility and MAR aspects. The findings from our interviews demonstrated that parenthood is still gender related ( Gurkan, 2021; Paschal *et al.*, 2011). In our study, some participants perceived that bigger burden falls on mothers because they consider that the *essentialisation of motherhood* is imposed by traditional gender roles and pro-birth important policies ( Tarkhanova, 2018).

Other young participants, however, seemed to have expectations that father’s role is to provide financial support or is considered as a breadwinner in general. This seemed particularly true for most young participants in the study sample, suggesting how best to. The interviewed participants in the study, considered male (in)fertility as a taboo in their social networks, not much discussed in public. The taboo surrounding male infertility illustrates how pronatalist discourses not only privilege reproductive success but also actively obscure and delegitimise men’s reproductive vulnerability.

The descriptive findings regarding (in)fertility reveal a deeper social logic where reproductive success is conflated with adulthood and social maturity ( Boivin *et al.*, 2007; Facchin *et al.*, 2020). This is particularly salient in the post-socialist context Szalma’s (2021, where the retreat of the state from welfare provision has reinforced the family as the primary site of security. Therefore, young people’s reproductive aspirations are not just cultural artifacts but are strategically embedded in a moral economy where children represent social capital and future security. Theorizing these views as responses to structural precariousness allows us to see how 'tradition' is often a pragmatic reaction to the lack of institutional support for work-family reconciliation.

The findings suggest that infertility in Kosovo is not experienced as a uniform medical condition but is filtered through stratified gender expectations that reflect a broader post-socialist gender regime. For the female participants, infertility is framed as a failure of social citizenship and a threat to their primary status within the kin group. This 'a priori' blame placed on women is a manifestation of a moral economy where a woman’s social value is inextricably tied to her reproductive output ( Cicerchia, 2023). Consequently, the social pressure to conceive—particularly in rural areas—is not merely a cultural preference but a normative framework that enforces female domesticity.

In contrast, the limited awareness and reluctance to discuss male infertility among participants reveals a social logic designed to protect hegemonic masculinity. For men, infertility is experienced less as a social utility failure and more as a crisis of virility and 'ego.' By rendering male infertility socially invisible, the prevailing power relations shift the entire 'burden of proof' and the accompanying stigma onto women. This distinction creates a gendered power dynamic where women’s infertility becomes a public family crisis, while male infertility remains a private, silenced shame.

These divergent social meanings deeply shape how each gender engages with, or avoids, Medically Assisted Reproduction (MAR) information. Our results nuance the findings of Vialle *et al.* (2023) and Schick *et al.* (2016) by demonstrating that 'knowledge' about infertility is not neutral; it is an active negotiation within a gendered landscape where silence and stigma serve to maintain traditional status hierarchies in a precarious post-socialist environment.

A substantial lack of knowledge was found in relation to MAR techniques, highlighting the importance of detailed information about the whole process of MAR treatments. A total of 5 private MAR clinics are currently based in Kosovo. Information provided on their websites was implicitly aimed toward heterosexual couples and conventional family models experiencing infertility; there was no mention of LGTB individuals or single women whatsoever (they are not permitted to access). Women were given greater attention than men, there is no any information regarding access requirements, such as age restrictions or how to obtain access. The accounts revealed that interviewed participants were fully aware of existence of MAR techniques, however, the details and type of right synthesized information are deficient to avoid further confusion with people. Although, we did identify various gaps in understanding the MAR process discussed above, it is the health authorities along with MAR clinic that provide suitable information and thus it is essential that the persons who are seeking fertility treatment should be informed of available alternatives to MAR. Due to the fact that the majority of MAR clinics are private, financial and economic factors are among the barriers found in our research that have been recognized to affect access to MAR clinics but have not been extensively examined in the context of fertility. Majority of participants have stressed that the biggest problem in accessing such services is financial incapacity of the population, as these services are not covered and should be paid out of pocket. Our study serves as an example how private MAR clinics decrease the access to people who want to conceive with alternative methods because of the increased costs.

## Conclusion

This study is the first of its kind in South-East Europe and its results are useful for domestic healthcare stakeholders and policy-makers. The majority of male and female respondents wished to have children and they had an overall positive attitude towards perceptions of parenthood, but contingent upon having a favourable socio-economic situation. Perceptions about infertility have a clear gender component, being considered a problem that particularly affects women and revealing taboos associated with male infertility. Our findings indicate that there is a clear gap of knowledge and relevant need for information about (in)fertility, MAR methods to the young people in Kosovo.

Beyond a descriptive summary of views, this study demonstrates how reproductive attitudes are shaped by the tensions of post-socialist transition. The 'persistent' pronatalist norms identified among Kosovar youth are not static remnants of the past; they are actively reconstructed within a contemporary moral economy ( Cicerchia, 2023) that grapples with migration, economic uncertainty, and shifting gender roles. By situating MAR perceptions ( Szalma, 2021), within these broader gender regimes, we uncover how power operates through 'normative' frameworks that pressure individuals to conform to specific reproductive timelines while simultaneously offering little structural support for those who deviate from them.

This regional perspective underscores the need for context-sensitive educational and policy initiatives that address both structural and cultural barriers to reproductive health knowledge and equality.

In our views, it is recommended that health authorities create a catalogue of informed consent forms and information sheets that comply with current regulations in Kosovo. Persons seeking fertility treatment should be informed of available alternatives to MAR, including adoption and the possibility of treating infertility through biomedical intervention.

## Limitations

The sample was primarily drawn from Prishtina, the capital of Kosovo, which serves as the nation’s primary hub for higher education and internal migration. While this provides a rich look at an urbanized, educated demographic, the geographic concentration constitutes a limitation regarding the generalizability of the findings. Specifically, the results may reflect an 'urban-educational' bias that does not fully capture the internal heterogeneity of Kosovar society. In more rural or economically marginalized regions, the moral economy of reproduction may be even more rigid, and patrilineal power relations more pronounced, due to the relative absence of the diverse social discourses present in the capital.

## Ethical approval and consent to participate

This project (n°2021.004) received ethical approval from the Research Ethics Committee of the University of Navarra on 29 January 2021. The Research Ethics Committee reviewed the Protocol, Patient Information sheet and Informed Consent dated 26/01/2021. On 29/02/2021 the Committee issued a statement for the realization of the project since it has considered that it conforms to the essential ethical principles and the deontological principles of the Institutions.

All interview participants provided informed consent. For in-person interviews, consent forms were signed physically, while for online interviews, the forms were sent as PDFs and returned signed by participants. The consent form indicated that transcripts would be used for preparing scientific articles and reports related to B2-InF. Additionally, participants provided audio-recorded consent at the beginning of each interview.

## Data Availability

The data underlying these results cannot be shared due to confidentiality concerns. The sensitive nature of the information poses a risk of exploitation by the fertility industry for commercial purposes. To address these concerns, the Grant Agreement (nr. 872706 — B2-InF) signed between the Research Executive Agency of the European Commission and all B2-InF project partners specifies that the dissemination level of the interviews, as well as the corresponding reports and thematic analyses, is classified as "confidential." This designation is explicitly defined as "accessible only to members of the consortium (including the Commission Services)" To request access, please email the corresponding author. Access will be granted after verifying that the data will be used exclusively for scientific research purposes and that all researchers involved in the study have no conflicts of interest.

## Software availability statement

The qualitative analysis in this article was performed using Atlas.ti (version 23.2.1), a proprietary qualitative data analysis software. For more information about Atlas.ti, visit the Atlas.ti website www.atlasti.com. For those without access, equivalent analyses can be performed using open-source alternatives such as Taguette ( https://www.taguette.org/).

## Appendix

**Themes and sample questions:**

- 1. **Parenthood and Childlessness**
  - What do you think about parenthood?
  - How is childlessness perceived in your context?
  - Are there gendered implications of voluntary vs. involuntary childlessness?
- 2. **(In)Fertility**
  - How relevant is fertility/infertility in your social context?
  - Are there taboos or public discussions around these topics?
- 3. **MAR Treatments and Expectations**
  - What do you know about available reproductive services?
  - Would you consider using MAT in the future?
  - What ethical concerns do you associate with MAR?
- 4. **Access and Legal Framework**
  - How accessible are MAR services in your country?
  - What do you know about legal regulations and their impact on different family types?
- 5. **Public Information and Trust**
  - Where do you get information about MAR?
  - Is the information trustworthy and inclusive?
  - Who should be the target audience for MAR-related communication

## References

[ref-31] Apostolovska Toshevska B Madjevikj M Ljakoska M : Attitudes towards reproduction and creating a family among Albanian Women—the case of Arachinovo municipality, North Macedonia.In: Zafeiris, K.N., Kotzamanis, B., Skiadas, C. (eds) *Population Studies in the Western Balkans. European Studies of Population. *Springer, Cham,2024;26. 10.1007/978-3-031-53088-3_16

[ref-1] Archer NP Langlois PH Suarez L : Association of paternal age with prevalence of selected birth defects. *Birth Defects Res A Clin Mol Teratol.* 2007;79(1):27–34. 10.1002/bdra.20316 17120236

[ref-2] ATLAS.ti Scientific Software Development GmbH: ATLAS.ti Mac (version 23.2.1). Qualitative data analysis software,2023. Reference Source

[ref-30] Boivin J Bunting L Collins JA : International estimates of infertility prevalence and treatment-seeking: potential need and demand for infertility medical care. *Hum Reprod.* 2007;22(6):1506–1512. 10.1093/humrep/dem046 17376819

[ref-3] Brunborg H : Report on the size and ethnic composition of the population of Kosovo. Case of Slobodan Milosevic (IT-02-54), ICTY, La Haye,2002. Reference Source

[ref-34] Cicerchia L : Marx, Malthus, and the moral economy of reproduction. *Hypatia.* 2023;38(3):587–606. 10.1017/hyp.2023.69

[ref-4] Čipin I Zeman K Međimurec P : Cohort fertility, parity progression, and family size in former Yugoslav countries. *Comparative Population Studies.* 2020;45. 10.12765/CPoS-2020-18

[ref-5] Cleary-Goldman J Malone FD Vidaver J : Impact of maternal age on obstetric outcome. *Obstet Gynecol.* 2005;105(5 Pt 1):983–990. 10.1097/01.AOG.0000158118.75532.51 15863534

[ref-6] Daniluk JC : Reconstructing their lives: a longitudinal, qualitative analysis of the transition to biological childlessness for infertile couples. *J Couns Dev.* 2001;79(4):439–449. 10.1002/j.1556-6676.2001.tb01991.x

[ref-7] Facchin F Leone D Tamanza G : Working with infertile couples seeking assisted reproduction: an interpretative phenomenological study with infertility care providers. *Front Psychol.* 2020;11: 586873. 10.3389/fpsyg.2020.586873 33391106 PMC7773748

[ref-8] Frejka T Gietel-Basten S : Fertility and family policies in Central and Eastern Europe after 1990. *Comparative Population Studies.* 2016;41(1):3–56. 10.12765/CPoS-2016-03en

[ref-9] Gurkan T : A qualitative study on the perception of fatherhood. *Eur J Educ Sci.* 2021;8(2):42–59. 10.19044/ejes.v8no2a42

[ref-10] Kaser K : Patriarchy after patriarchy: gender relations in Turkey and in the Balkans, 1500–2000. Lit Verlag,2008. Reference Source

[ref-32] Kontogiannis G : The evolution of family-related behaviours in the Western Balkans and their impact on present and future family and population structures: an analysis concerning the period 1945–2050.In: Zafeiris, K.N., Kotzamanis, B., Skiadas, C. (eds) *Population Studies in the Western Balkans. European Studies of Population.*Springer, Cham,2024;26. 10.1007/978-3-031-53088-3_5

[ref-11] Krasniqi M : The role of tradition in self-reproductive knowledge among Albanians. *Albanological Traces - Folklore and Ethnology.* 1990;19.

[ref-12] Latifi T : Family, kinship relations and social security in Kosovo since the war of 1999. Doctoral dissertation, Graz,1999. Reference Source

[ref-14] Lerch M : Fertility and union formation during crisis and societal consolidation in the Western Balkans. *Popul Stud (Camb).* 2018;72(2):217–234. 10.1080/00324728.2017.1412492 29357746

[ref-15] Paschal AM Lewis-Moss RK Hsiao T : Perceived fatherhood roles and parenting behaviors among African American teen fathers. *J Adolesc Res.* 2011;26(1):61–83. 10.1177/0743558410384733

[ref-16] Predojević J : Fertility transition in works of Albanian authors from Kosovo and Metohia. *Stanovnistvo.* 2001;39(1–4):131–156. 10.2298/STNV0104131P

[ref-17] Pushka A : Factors of birth rate and their quantification. *Problems of population renewal policy in Serbia.* 1989.

[ref-18] Regushevskaya E Hemminki E Klemetti R : Postponing births - comparing reasons among women in St Petersburg, Estonia, and Finland. *Finnish Yearbook of Population Research, XLVIII. * 2013;127–145. Reference Source

[ref-35] Sahoo S Das A Dash R : The psychological impact of male infertility: a narrative review. *Cureus.* 2025;17(8): e89453. 10.7759/cureus.89453 40918879 PMC12413911

[ref-19] Sartorius GA Nieschlag E : Paternal age and reproduction. *Hum Reprod Update.* 2010;16(1):65–79. 10.1093/humupd/dmp027 19696093

[ref-13] Schick M Rösner S Toth B : Effects of medical causes, role concepts and treatment stages on quality of life in involuntary childless men. *Andrologia.* 2016;48(9):849–854. 10.1111/and.12519 27739143

[ref-20] Schmidt L Sobotka T Bentzen JG : Demographic and medical consequences of the postponement of parenthood. *Hum Reprod Update.* 2012;18(1):29–43. 10.1093/humupd/dmr040 21989171

[ref-21] Shiffman J Skrabalo M Subotic J : Reproductive rights and the state in Serbia and Croatia. *Soc Sci Med.* 2002;54(4):625–642. 10.1016/s0277-9536(01)00134-4 11848279

[ref-22] Struyf J Hens K Rozée V : Gender aspects in parenthood conceptions of young Europeans. A critical reflection on the European Project B2-InF [version 1; peer review: 2 approved with reservations]. *Fam Relatsh Soc.* [Forthcoming],2023. 10.12688/openreseurope.18210.1

[ref-26] Szalma I : Attitudes, norms, and beliefs related to assisted reproduction technologies among childless women in a pronatalist society. *Springer Fachmedien Wiesbaden. * 2021. 10.1007/978-3-658-35628-6

[ref-23] Tarkhanova O : Essentializing motherhood: the Ukrainian woman in policy debates. *InterDisciplines: Journal of History and Sociology.* 2018;9(1). 10.4119/indi-1053

[ref-24] The World Bank: Fertility rate, total (births per woman) - Switzerland.2023; Retrieved October 15, 2023. Reference Source

[ref-33] Vasić PD : Fertility postponement between social context and biological reality: the case of Serbia. *Sociológia-Slovak Sociological Review.* 2021;53(3):309–336. 10.31577/sociologia.2021.53.3.12

[ref-25] Vialle M Struyf J Hens K : Between acceptance and rejection: young Europeans' ambivalent relationship with the procreative norm. *J Gend Stud.* [Forthcoming],2023.

