# Supplementary material for: Exploring MAR perceptions in Kosovo – disparities in knowledge about MAR among young people
Source: Open Res Eur. 2026 Apr 2;5:20. Originally published 2025 Jan 22. [Version 5] doi: 10.12688/openreseurope.18974.5 (PMC12906643; doi:10.12688/openreseurope.18974.5)
Supplement: Peer review history

# Reviewer report: Reviewer response for version 4

**DOI:** 10.21956/openreseurope.24782.r68741

**Published:** 2026-02-14

**Authors:** Karen P Phillips

**Affiliations:** 1 University of Ottawa,, Ottawa, Canada

I understand that this manuscript has been reviewed and revised previously. This is my first review of this manuscript.

 I note that MAR was adopted by the authors/previous reviewers, however it is non-standard in many countries. If standard in the region this is acceptable, I defer to the authors but ART should be defined as well for the international reader.

 I note some English syntax/grammatical issues in the manuscript- these need to be reviewed.

 Introduction- Authors establish that fertility trends have affected the Western Balkan region, resulting in fewer children/couple, but present as evidence pronatalist culture, lack of birth control (all of which would be presumed to increase fertility rate), and then posit a series of factors which affect fertility rate in an unclear manner e.g. political (presumed pessimism would decrease fertility rate), low female education/economic activity typically would increase fertility rate,  ‘persistent agrarian settlement’ is used as potential deterrent of parenthood, but is not explained and is not evident.

 The paragraph ‘(in)fertility patterns- should be moved up one paragraph or joined with previous paragraph- seems disjointed. Further changes to demographic patterns do not necessarily indicate infertility, so this word should be removed.

 The context about MAR- legal frameworks, policies, scope should (if possible) be referenced. These references could include online documents, websites, Ministry of Health website etc.

 Methods- needs subheadings to better organize the components. The first section of the Methods incorporates recruitment, study design, participant selection, ethics and a bit of data analysis, and then the next section is called ‘Data Analysis’. The Methods also includes the Demographics table (move to Results) and the major themes (move to Results).

 Response to reviewers is embedded in the manuscript text- this needs to be removed: “We acknowledge that this recruitment approach may have favoured individuals with higher education or greater urban engagement, and that the relatively small sample size limits generalisability. These clarifications have been added to the Methods section to improve transparency and to contextualise the scope and  potential limitations of the study.”  Limitations should appear in a separate section towards the end of the Discussion.

 Was the interview guide informed by other instruments? Existing literature? Pilot tested?

 The selection criteria is unclear. Age range is consistent, but references to the B2-InF project indicate objective is to ‘to explore how young, childless individuals—those who have not yet actively considered parenthood or encountered fertility challenges’, however the inclusion criteria does not exclude parenting respondents, nor does it appear to have precluded previous pregnancy experiences (if these did not result in live birth). Study objective appears later in Methods : ‘Therefore, this study provides an opportunity to gain insight into fertility behaviour and reproduction as they capture views of young people who are not parents about motherhood/fatherhood and (in)fertility.’

 Data analysis- thematic analysis requires reference. Suggest alignment with established qualitative approaches eg. Equator SRQR https://www.equator-network.org/reporting-guidelines/srqr/

 Results

 ‘transgender female to male’ may be written trans man, ideally the participant gender identity was verbalized by the participant with English translation KOS 03- presumably ‘None’ refers to no identified religion? I would remove this word as it is confusing

 The authors have included a bit of discussion text at the end of the first theme section, this is unusual. Not familiar with the formatting of the journal, but either (1) have distinct Results and

 Discussion sections, or (2) have a combined Results and Discussion section. The latter would require significant reformatting.

 ‘Many participants agreed that infertility is increasing, with both men and women experiencing difficulties, albeit in different ways.’ Was the idea that infertility was increasing stated to participants? How did this agreement emerge? It is unclear how the quotes align with the interview guide, some interjection of the scope of the question would be helpful.

 Discussion

 The text is well written. Given the diversity of the sample with respect to gender identity, religion and sexual orientation, I would have expected a bit more discussion as to the intersection of these characteristics and parenting intentions, access to MAR. In contrast, the discussion of binary gender (male/female) and (in)fertility is well presented.

 The authors are encouraged to further engage with literature to help contextualize the findings with respect to the respondents using a generation Z lens, although there is substantial Kosovo context provided.

 Limitations would have to include heterogeneity of the sample, and lack of apparent data on prior conceptions/pregnancies as this is central to the perception of fertility/parenting intentions.

If applicable, is the statistical analysis and its interpretation appropriate?

Not applicable

Is the study design appropriate and is the work technically sound?

Partly

Is the work clearly and accurately presented and does it engage with the current literature?

Partly

Are the conclusions drawn adequately supported by the results?

Partly

Are sufficient details of methods and analysis provided to allow replication by others?

Partly

Are all the source data and materials underlying the results available?

No source data required

Reviewer Expertise:

sexual and reproductive health

I confirm that I have read this submission and believe that I have an appropriate level of expertise to confirm that it is of an acceptable scientific standard, however I have significant reservations, as outlined above.

# Reviewer report: Reviewer response for version 3

**DOI:** 10.21956/openreseurope.23626.r63793

**Published:** 2026-01-07

**Authors:** Catarina Delaunay

**Affiliations:** 1 Universidade NOVA de Lisboa, Lisbon, Portugal

I would like to commend the authors for the substantial improvements made in this revised version of the manuscript, which now presents a more coherent analysis of young people’s perceptions of fertility, infertility, and medically assisted reproduction (MAR) in Kosovo. Although I was not involved in the assessment of earlier versions, it is clear that significant effort has been invested in revising the paper in response to previous feedback. The current version demonstrates clearer conceptual framing and a more consistent engagement with relevant scholarship, resulting in improved overall coherence. The attempt to situate the findings within broader debates on pronatalism, gender norms, and reproductive governance in Central and Eastern Europe is a valuable step forward.

 Nevertheless, some important issues remain that merit further attention before the paper can be considered fully ready for indexing. These concerns are outlined below in general and specific comments.

 General comments, numbered:

 1. Background and literature review

 While the engagement with the literature has been strengthened, it remains somewhat limited, and further integration of up-to-date and diverse scholarly perspectives would enhance the analytical depth and contextual grounding of the study. More specifically, the discussion of women’s subordinate position in family and reproductive life in Kosovo relies predominantly on literature from the 1980s, 1990s, and early 2000s. While these works are foundational, the manuscript would benefit from a more explicit reflection on the extent to which these patriarchal norms persist today, and on how their manifestations may vary across regions (e.g., urban versus rural contexts) and socioeconomic settings. Without this, there is a risk of portraying gender relations in Kosovo as static or insufficiently contextualised historically, given that only a single study from Hungary is used to frame the analysis of the current situation in terms of gender norms and fertility behaviors in pronatalist societies. I therefore suggest consulting two chapters from the volume cited below, namely “The Evolution of Family-Related Behaviours in the Western Balkans and Their Impact on Present and Future Family and Population Structures: An Analysis Concerning the Period 1945–2050” and “Attitudes Towards Reproduction and Creating a Family Among Albanian Women—The Case of Arachinovo Municipality, North Macedonia.”

 Zafeiris KN, Kotzamanis B, Skiadas C (eds.): Population Studies in the Western Balkans. Springer Cham. European Studies of Population 26. 2024.

 Other relevant papers worth consulting for this contextual framing include the following:

 (Refer ref no.1) &

 Vasić PD: Fertility Postponement between Social Context and Biological Reality: The Case of Serbia. Sociológia - Slovak Sociological Review. 2021; 3: 309–336.

 When describing the legal regulation of MAR in Kosovo, the manuscript should also specify the conditions of access to treatment. In particular, it should be clearly stated whether MAR services are publicly funded, partially covered by health insurance, or entirely paid out of pocket. This information currently appears only later in the results section through participants’ narratives, but it would strengthen the contextual framing to address it earlier in the background section.

 2. Methodological transparency and sampling procedures

 While the manuscript provides a general description of the study design and the characteristics of the sample, it still lacks sufficient detail regarding the recruitment process and sampling strategy. While the authors state that purposive sampling was used to capture sociocultural heterogeneity (in terms of gender, age, residence, marital status/relationship, sexual orientation, education, and religion), it remains unclear how participants were identified, approached, and selected. In particular, the manuscript would benefit from clarifying through which channels participants were recruited (e.g., personal networks, institutions, social media, community organisations) and whether recruitment strategies may have favoured certain socio-demographic profiles or viewpoints. This issue is of particular importance given that the majority of the interviewees were recruited primarily from Prishtina, the capital city of Kosovo. Given the country’s demographic diversity and population size, further clarification is necessary to assess the scope, potential biases, and limitations of the study. Therefore, the claim of heterogeneity should be better substantiated, and the limitations deriving from the relatively small sample size should be more explicitly acknowledged. Providing this information would substantially improve the methodological transparency of the study and allow readers to more accurately assess the robustness of the findings.

 Moreover, the description of the data analysis would benefit from greater clarity and precision. While the authors outline several analytical steps, the overall process remains difficult to follow. It is unclear how coding was conducted in practice, who was involved at each stage, and how responsibilities were distributed between the local researcher and the broader research team. The text states that codes were “pre-defined,” then later that an “initial code structure” was developed from the data, and subsequently that themes were “inductively derived.” These statements suggest different analytical logics (deductive vs. inductive) that are not sufficiently reconciled. The authors should clarify whether the analysis followed a primarily inductive, deductive, or hybrid approach, and how this was operationalised in practice. The description of thematic development is also somewhat repetitive and would benefit from clearer distinctions between codes, themes, and analytical levels.

 In addition, given that the key thematic areas of the interview guide are described in the main text, the manuscript would benefit from moving the list of sample questions to an appendix, allowing the main text to focus more clearly on analytical issues.

 3. Conceptual precision, analytical depth, and interpretation

 While the manuscript engages with important themes, there are several areas where greater conceptual precision would strengthen the analysis. At times, concepts such as “tradition,” “culture,” and “religion” are invoked in a relatively broad or taken-for-granted manner. Treating these notions as self-evident risks reifying them, rather than analysing them as socially constructed, historically contingent, and internally heterogeneous. Greater conceptual refinement in this respect would enhance the analytical depth of the study.

 The empirical material presented is rich and offers valuable insights into young people’s perceptions of (in)fertility, parenthood, and assisted reproduction. However, the analysis frequently remains at a descriptive level, summarising participants’ views without fully interrogating the underlying social logics and normative frameworks that shape them. A stronger analytical contribution would involve more explicitly theorising why these views persist, how they are embedded in power relations, and how they relate to broader gender regimes and moral economies of reproduction in post-socialist contexts.

 Relatedly, although the manuscript draws on relevant literature—particularly on pronatalism and gender norms—these references are sometimes used in a largely illustrative manner. The analysis would benefit from a more explicit dialogue with this scholarship, highlighting not only points of convergence but also areas where the findings complicate, nuance, or diverge from existing studies, particularly in relation to the specificities of the Kosovar context.

 The discussion of gendered perceptions of parenthood would also benefit from greater analytical precision. While the manuscript notes that motherhood is often perceived as a more demanding role than fatherhood, involving a stronger emotional and biological bond and the burden of raising the child, the two illustrative quotations used to support these claims are drawn from male participants. It remains unclear whether this perception is shared equally across genders or whether it differs between male and female respondents. Given that gender constitutes a central analytical axis of the study, a more systematic comparison of male and female perspectives would substantially strengthen the analysis. Similarly, when stating that infertility is experienced differently by men and women, the manuscript should more clearly articulate how these differences manifest and what social meanings they carry, rather than presenting them in largely descriptive terms.

 Finally, in the Discussion section, the heterogeneity of the sample is presented as a strength of the study. Given the relatively small sample size and the recruitment strategy employed, this claim would benefit from greater caution. Rather than framing heterogeneity as a strength in itself, it would be more appropriate to acknowledge it as a partial and limited characteristic of the sample, with implications for the scope and generalisability of the findings.

 4. Presentation and clarity

 The manuscript is generally well written, but some sections would benefit from further linguistic tightening to reduce repetition and enhance readability. Additionally, minor inconsistencies in phrasing and occasional awkward formulations remain and could be addressed through careful copy-editing. Some of these minor issues are listed below.

 Specific comments, by page number:

 On pages 1–2 (Abstract), the sentence referring to “the importance of (in)fertility” is conceptually awkward. Rather than framing fertility and infertility in terms of “importance,” it would be more precise to refer to how they are perceived, understood, or socially constructed. For instance, the sentence could be reformulated as: “...analysing how (in)fertility is perceived by young people from a gendered perspective.” This awkward formulation also appears in the main text, as one of the themes and subtitles. A possible option could be “the perceived impact of (in)fertility.”

 On page 3, the first paragraph is missing final punctuation.

 On page 3, in the sentence “While in 1991 the average number of children per woman in Kosovo was 3.25 (Brunborg, 2002), by 2019 this figure had declined to 1.97…”, the source for the 2019 figure must be explicitly cited. Is it World Bank, 2023? In addition, the extra full stop at the end of the sentence should be removed.

 On page 3, in the sentence referring to “Kosovo’s secular tradition of customary law,” the placeholder symbols (“???”) must be deleted.

 On page 3, please ensure that the acronym MAR is written in full at its first mention and used consistently thereafter, avoiding alternation with ART.

 On page 5 (Table 1), the heading “Demographic characteristic of participants” should be corrected to “Demographic characteristics of participants.” In addition, the category currently labelled “Transgender male to female” should be revised to “Transgender female to male,” to remain consistent with how this identity is described in the interview excerpts.

 On page 5, the sentence “Planning the parenthood participants is considered when they become fully grown persons…” requires revision for clarity and grammatical correctness.

 On page 6, the sentence “Participants don’t think a woman’s capacity to conceive will change after she reaches 30;rather, they view fertility as a gift as long as a woman is capable of bearing children” contains a typographical error. A space is missing before the word “rather.”

 On page 7, the sentence currently reading “Many participants agreed that infertility is increasing where men and women struggle equally for being infertile” should be revised for clarity. A suggested reformulation is: “Many participants agreed that infertility is increasing, with both men and women experiencing difficulties, albeit in different ways.”

 On page 7, when listing the possible causes of infertility mentioned by participants, the expression “healthy lifestyles” appears to be incorrect and should likely read “unhealthy lifestyles.”

 On page 7, please ensure that the acronym IVF is written in full at its first occurrence.

 On page 7, the interview excerpt ending with “(KOS 11, 22 years, female, bisexual, Muslim))” contains an extra parenthesis that should be removed.

 On page 7, the sentence “Another aspect of MAR in Kosovo during the interviews was the legal issues and what is the general knowledge” is grammatically unclear and should be reformulated, for example: “Another aspect of MAR that emerged from the interviews relates to legal issues and participants’ level of knowledge about them.”

 On page 9 (Conclusion), there is a typographical and conceptual error in the sentence stating that taboos are associated with “female infertility.” Based on the analysis presented, this should read “taboos associated with male infertility.”

 Overall, the paper addresses an important and under-researched topic and makes a valuable contribution to discussions on reproductive norms and medically assisted reproduction in Kosovo. With greater transparency regarding recruitment procedures and a more analytically developed engagement with the data, the manuscript would constitute a solid contribution to scholarship on reproductive governance, gender, and family formation in contemporary Europe.

If applicable, is the statistical analysis and its interpretation appropriate?

Not applicable

Is the study design appropriate and is the work technically sound?

Partly

Is the work clearly and accurately presented and does it engage with the current literature?

Partly

Are the conclusions drawn adequately supported by the results?

Yes

Are sufficient details of methods and analysis provided to allow replication by others?

Partly

Are all the source data and materials underlying the results available?

Yes

Reviewer Expertise:

My research interests include the sociology of the family, the sociology of gender, the sociology of health and medicine, and science and technology studies. For the past 15 years, I have focused on sociotechnical controversies surrounding medically assisted reproduction, including reproductive trajectories, fertility preservation, third-party reproduction, and processes of decision-making and meaning-making around IVF embryos.

I confirm that I have read this submission and believe that I have an appropriate level of expertise to confirm that it is of an acceptable scientific standard, however I have significant reservations, as outlined above.

## References

[1] : Son Targeting Fertility Behaviour in Albania. *The Journal of Development Studies* .2024;60(8) : 10.1080/00220388.2024.2354230 1246-1265 10.1080/00220388.2024.2354230

# Reviewer report: Reviewer response for version 3

**DOI:** 10.21956/openreseurope.23626.r64279

**Published:** 2025-12-04

**Authors:** Szalma Ivett

**Affiliations:** 1 Corvinus University of Budapest, Budapest, Hungary

In this third version, the authors have substantially strengthened the manuscript by expanding the regional and theoretical background, clarifying the methodology, and better situating the Kosovar case within broader Central and Eastern European debates on pronatalism, gender norms, infertility and MAR. The qualitative design is appropriate, the analysis is transparent, and the discussion now clearly links the findings to existing scholarship.

 In my previous round, I asked the authors to use the term “Medically Assisted Reproduction (MAR)” consistently, in line with the title. In this version, the terminology is largely harmonized, but there are still a few occurrences of “ART” in the text. I recommend a final language edit to standardize the terminology (preferably using MAR throughout, with a brief clarification sentence when the term first appears). I also kindly ask the authors to correct the percentage for “Married” in Table 1 (N=5 out of 15 should be 33%, not 53%) and to check the World Bank reference label, as well as a few remaining minor language issues.

If applicable, is the statistical analysis and its interpretation appropriate?

Not applicable

Is the study design appropriate and is the work technically sound?

Partly

Is the work clearly and accurately presented and does it engage with the current literature?

Partly

Are the conclusions drawn adequately supported by the results?

Yes

Are sufficient details of methods and analysis provided to allow replication by others?

Partly

Are all the source data and materials underlying the results available?

Yes

Reviewer Expertise:

NA

I confirm that I have read this submission and believe that I have an appropriate level of expertise to confirm that it is of an acceptable scientific standard.

# Reviewer report: Reviewer response for version 2

**DOI:** 10.21956/openreseurope.22116.r60145

**Published:** 2025-10-01

**Authors:** Szalma Ivett

**Affiliations:** 1 Corvinus University of Budapest, Budapest, Hungary

Review of the Article

 The manuscript provides an important and timely contribution by exploring perceptions of parenthood, fertility, infertility, and assisted reproduction (MAR) among young people in Kosovo. It is the first of its kind in this context, and the authors should be commended for addressing a topic that has remained under-researched in the Western Balkans despite its significant societal and policy relevance. The paper highlights key themes around the persistence of traditional gender norms, the taboo surrounding male infertility, and the general lack of knowledge about MAR—findings that are highly relevant both locally and in comparative perspective.

**Strengths** of the study include its exploratory design, the focus on a largely neglected population, and the careful thematic analysis. The authors also succeed in connecting their results to wider debates on parenthood and fertility in Europe, which increases the manuscript’s broader relevance.

 However, there are also several **limitations and areas for improvement** that should be addressed:

1. **Sample size and composition** – The study is based on only 15 interviews. While qualitative research does not require large samples, the authors should justify why this particular number was considered sufficient to reach saturation. In addition, the decision to limit the age range to 18–30 requires clearer reasoning. Why were older childless adults, who might also face issues of infertility and pronatalist pressure, excluded? Addressing these points would strengthen the methodological transparency.
2. **Interview guide** – The article mentions the use of a semi-structured interview technique but does not provide the interview guide or even a summary of the main questions. Including the full guide as an appendix (or at least outlining the key themes and sample questions) would improve replicability and allow readers to better assess the quality and focus of the data collection.
3. **Theoretical grounding and references** – While the study draws on some literature from the Western Balkans and beyond, it could be significantly enriched by engaging with additional international scholarship. In particular, I strongly recommend citing:
  - Szalma, I. (2021). *Attitudes, Norms, and Beliefs Related to Assisted Reproduction Technologies among Childless Women in a Pronatalist Society*. Springer Fachmedien Wiesbaden. https://doi.org/10.1007/978-3-658-35628-6
  This book is directly relevant, as it discusses assisted reproduction in a pronatalist society, the role of traditional gender norms, the taboo of male infertility, and the knowledge gap surrounding MAR—precisely the themes the current article highlights in the Kosovar context. By engaging with this reference, the authors could situate their findings within a broader Central and Eastern European scholarly discourse and strengthen the comparative dimension of their analysis.
4. **Clarity and style** – The manuscript would benefit from further editing for language and style, as some sections contain repetitive or awkward phrasing. Improving readability will make the contribution more accessible to an international audience.

**Conclusion**: Overall, the manuscript addresses an original and highly relevant topic. With revisions that clarify methodological choices, provide more transparency about the data collection tools, and engage with additional literature , the article could make a valuable contribution to scholarship on fertility, gender norms, and reproductive technologies in Southeastern Europe. I recommend indexing subject to these revisions.

If applicable, is the statistical analysis and its interpretation appropriate?

Not applicable

Is the study design appropriate and is the work technically sound?

Partly

Is the work clearly and accurately presented and does it engage with the current literature?

Partly

Are the conclusions drawn adequately supported by the results?

Yes

Are sufficient details of methods and analysis provided to allow replication by others?

Partly

Are all the source data and materials underlying the results available?

Yes

Reviewer Expertise:

My areas of research include sociology of family, fertility and reproductive decision-making, childlessness, and gender norms. I also work on topics related to assisted reproduction, pronatalist policies, and attitudes toward infertility in Central and Eastern Europe.

I confirm that I have read this submission and believe that I have an appropriate level of expertise to confirm that it is of an acceptable scientific standard, however I have significant reservations, as outlined above.

## References

[1] : Attitudes, Norms, and Beliefs Related to Assisted Reproduction Technologies among Childless Women in a Pronatalist Society. 10.1007/978-3-658-35628-6 10.1007/978-3-658-35628-6
